# Construction of a Lactate-Related Prognostic Signature for Predicting Prognosis, Tumor Microenvironment, and Immune Response in Kidney Renal Clear Cell Carcinoma

**DOI:** 10.3389/fimmu.2022.818984

**Published:** 2022-02-17

**Authors:** Zhuolun Sun, Wen Tao, Xudong Guo, Changying Jing, Mingxiao Zhang, Zhenqing Wang, Feng Kong, Ning Suo, Shaobo Jiang, Hanbo Wang

**Affiliations:** ^1^ Department of Urology, Third Affiliated Hospital of Sun Yat-sen University, Guangzhou, China; ^2^ Department of Urology, Minimally Invasive Surgery Center, The First Affiliated Hospital of Guangzhou Medical University, Guangzhou, China; ^3^ Department of Urology, Shandong Provincial Hospital Affiliated to Shandong First Medical University, Jinan, China; ^4^ Institute of Diabetes and Regeneration, Helmholtz Zentrum München, German Research Center for Environmental Health, Neuherberg, Germany; ^5^ Department of Urology, The First Affiliated Hospital of Sun Yat-sen University, Guangzhou, China

**Keywords:** kidney renal clear cell carcinoma, lactate, nomogram, prognostic signature, tumor microenvironment

## Abstract

Kidney renal clear cell carcinoma (KIRC) is one of the most prevalent primary malignancies with high heterogeneity in the urological system. Growing evidence implies that lactate is a significant carbon source for cell metabolism and plays a vital role in tumor development, maintenance, and therapeutic response. However, the global influence of lactate-related genes (LRGs) on prognostic significance, tumor microenvironment characteristics, and therapeutic response has not been comprehensively elucidated in patients with KIRC. In the present study, we collected RNA sequencing and clinical data of KIRC from The Cancer Genome Atlas (TCGA), E-MTAB-1980, and GSE22541 cohorts. Unsupervised clustering of 17 differentially expressed LRG profiles divided the samples into three clusters with distinct immune characteristics. Three genes (*FBP1*, *HADH*, and *TYMP*) were then identified to construct a lactate-related prognostic signature (LRPS) using the least absolute shrinkage and selection operator (LASSO) and Cox regression analyses. The novel signature exhibited excellent robustness and predictive ability for the overall survival of patients. In addition, the constructed nomogram based on the LRPS-based risk scores and clinical factors (age, gender, tumor grade, and stage) showed a robust predictive performance. Furthermore, patients classified by risk scores had distinguishable immune status, tumor mutation burden, response to immunotherapy, and sensitivity to drugs. In conclusion, we developed an LRPS for KIRC that was closely related to the immune landscape and therapeutic response. This LRPS may guide clinicians to make more precise and personalized treatment decisions for KIRC patients.

## Introduction

Renal cell carcinoma (RCC) represents one of the most common primary malignancies with increasing incidence in the urological system, constituting 2%–3% of adult malignant tumors (1). Kidney renal clear cell carcinoma (KIRC) accounts for ~80% of all the histological subtypes (2). Although most patients are diagnosed at the early stages, one-third of patients present with metastases at initial diagnosis, and a quarter develop metastases after therapy (3). Despite therapeutic advances in molecular targeted therapies, such as anti-vascular endothelial growth factors and mammalian target of rapamycin inhibitors, improving the overall survival (OS) and progression-free survival of patients remains a major clinical challenge (4). Therefore, exploring new potential markers for prognostic prediction and individualized treatment is of great clinical significance.

Lactate, historically viewed as a waste product of glycolysis and now also a significant carbon source for cell metabolism, plays a critical role in tumor development, maintenance, and therapeutic response (5, 6). KIRC is one of the tissues that highly rely on aerobic glycolysis and consequently produces a large amount of lactate under hypoxia, resulting in its accumulation in the tumor microenvironment (TME) (7, 8). Studies have demonstrated a close correlation between tumor lactate levels and tumor metastasis, recurrence, and poor prognosis (9, 10). Growing evidence is also showing a relationship between the upregulation of lactate dehydrogenase A (*LDHA*) and the proliferation of tumor cells. Using *in vitro* and *in vivo* experiments, Zhao et al. elucidated that *LDHA* upgrades RCC tissues and facilitates tumor migration and invasion through epithelial–mesenchymal transition (11). Moreover, an elevated level of *LDHA* can serve as a potential indicator of poor outcomes in patients with KIRC (12). Clinically, intervening metabolic pathways are attractive therapeutic targets with prognostic value for patients with cancers (13). In this regard, *LDHA* converts pyruvate into lactate and NAD^+^, as well as features in the metabolism of tumor cells by targeting c-Myc and hypoxia-inducible factor-1 (HIF-1), and is therefore considered as a promising anticancer target (6). Inhibitors of *LDHA*, such as *N*-hydroxyindole and galloflavin, can reduce cancer cell growth and invasion or induce apoptosis (14, 15). Moreover, the monocarboxylate transporter proteins (MCTs) are primarily engaged in lactate transport. MCT inhibitors such as AZD3965, AR-C155858, and α-cyano-4-hydroxycinnamate (CHC) reduce the levels of lactate in tumor cells *via* the inhibition of *SLC16A1* and *SLC16A7* (16, 17). Of these inhibitors, AZD3965 acquired encouraging preclinical success for advanced cancer in a phase I clinical trial (18). Thus, targeting lactate metabolism has been regarded as an exciting strategy for cancer therapy.

Immune checkpoint blockade (ICB), a frequently used immunotherapy, has demonstrated inspiring clinical success in multiple cancer types, but with low overall efficiency. In recent years, lactate has been proven to serve essential roles in immune response (19). Macintyre et al. revealed that effector T cells, especially cytotoxic T cells, become inactive under high lactate concentrations, subsequently resulting in the reduction of cytokine production and cell proliferation in both cancers and inflammatory diseases (20). In addition, another study reported that PKM2, a glycolytic enzyme that catalyzes phosphoenolpyruvate to pyruvate, exerts immunosuppressive functions *via* recruiting tumor-associated macrophages and myeloid-derived suppressor cells to the TME (21). Furthermore, lactate accumulation stimulated programmed death-ligand 1 (PD-L1) induction regulated by G protein-coupled receptor 81 (GPR81) in lung cancer cells, and knockdown of GPR81 reduced the level and activity of PD-L1 (22). Thus, it is necessary to estimate the immune landscape of KIRC to promote the development of immunotherapy and improve the outcomes of patients with cancer. The construction of a prognostic signature has been proven to be a feasible strategy to predict disease outcomes. To date, various risk signatures have been established to study the prognostic value of genes related to energy metabolism in KIRC. However, the roles of lactate-related genes (LRGs) in KIRC are still unknown.

In our study, we systematically analyzed the identified LRGs in patients with KIRC using The Cancer Genome Atlas (TCGA), E-MTAB-1980, and GSE22541 cohorts. Then, a lactate-related prognostic signature (LRPS) based on three genes was constructed to assess the prognosis of KIRC. Moreover, we analyzed the relationship of the LRPS with the immune microenvironment features and response to immunotherapy. These results may provide an alternative signature to predict the outcome and treatment efficacy in KIRC.

## Materials and Methods

### Dataset Source and Preprocessing

Publicly available expression and clinical data of patients with KIRC from TCGA (https://portal.gdc.cancer.gov/) were utilized as the training cohort in this study. The RNA sequencing data, mutation profiles, and corresponding clinical information were downloaded using the “TCGAbiolinks” package in R (23). In addition, the GSE22541 cohort (24) from the Gene Expression Omnibus (GEO) database (https://www.ncbi.nlm.nih.gov/) and the E-MTAB-1980 cohort (25) from the EMBL-EBI database (https://www.ebi.ac.uk/) were obtained as two independent validation cohorts. Batch effects were corrected using the ComBat function of the “sva” package in R (26). The detailed baseline clinical data of patients with KIRC are summarized in **Table 1**.

**Table 1:** Clinicopathological characteristics of the KIRC patients included in this study.

Variables	TCGA cohort	E-MTAB-1980 cohort	GSE22541 cohort
Age (years)			
≤65	347	57	–
>65	178	44	–
Gender			
Male	343	77	–
Female	182	24	–
Tissue			
Normal	72	0	0
Tumor	525	101	68
Grade			
1	17	13	–
2	229	60	–
3	205	23	–
4	74	5	–
Stage			
I	261	66	–
II	58	10	
III	124	13	–
IV	82	12	–

KIRC, kidney renal clear cell carcinoma; TCGA, The Cancer Genome Atlas.

### Collection of LRGs

The predefined gene sets included in our research were acquired from the Molecular Signatures Database (MSigDB; https://www.gsea-msigdb.org/gsea/msigdb/index.jsp) (27). We used the term “lactic” as the search keyword in the MSigDB database and eventually determined five priority LRG sets, namely, GOBP lactate metabolic process, HP increased serum lactate, HP lactic acidosis, HP lactic aciduria, and HP severe lactic acidosis. After deleting duplicates, 267 records were identified in total.

### Identification of Differentially Expressed LRGs and the Crosstalk Between These Genes

Principal component analysis (PCA) was first conducted to explore the separation between KIRC and non-tumor tissues using the expression data of LRGs. Differential expression of the LRGs was analyzed between the normal and tumor groups using the “limma” package in R, with *p* < 0.05 and |log_2_ fold change (FC)| ≥ 1.50. The protein–protein interactions (PPIs) among these differentially expressed genes (DEGs) were generated according to the STRING database (28) and visualized using Cytoscape v3.9.0 (29). The size of a node represents the number of direct interactions between nodes.

### Unsupervised Clustering of Differentially Expressed LRGs

Unsupervised clustering methods were applied to separate patients into distinct molecular subtypes based on the differentially expressed LRGs extracted from the TCGA and E-MTAB-1980 cohorts. A consensus clustering algorithm was conducted to identify the number of clusters and their stability using the “ConsensusClusterPlus” package in R (30). The R package ConsensuClusterPlus has been widely utilized in cancer-related studies (31–35). *K*-means clustering was conducted with 1,000 initial resampling and 50 iterations. The consensus matrix, cumulative distribution function (CDF), and relative change in area under the CDF curve were employed to select the best cluster number. The OS rates of the distinct clusters were assessed using Kaplan–Meier survival plots.

### DEGs between Distinct Clusters and Functional Enrichment Analysis

To further understand the pathways of the different clusters, we analyzed the expression of the DEGs between distinct clusters *via* the “limma” package in R, with significance set at *p* < 0.001. Gene Ontology (GO) annotation and Kyoto Encyclopedia of Genes and Genomes (KEGG) pathway enrichment analysis were performed with the WebGestalt database (http://www.webgestalt.org/) (36). Gene set enrichment analysis (GSEA) was utilized to analyze the functions related to the clusters according to the comprehensive gene expression profiles (37). A gene set with *p* < 0.05 and a false discovery rate (FDR) <0.25 was considered significantly enriched. The series of gene sets for marking 23 immune cell types was obtained from a previous study (38). The single-sample GSEA (ssGSEA) algorithm was then utilized to estimate the infiltration of immune cells in each sample with the “GSVA” package in R.

### Construction and Validation of the LRPS

Subsequently, the differentially expressed LRGs were subjected to univariate Cox regression analysis to determine genes related to OS using the data from TCGA. Statistically significant variables (*P* < 0.01) were then used for the least absolute shrinkage and selector operation (LASSO) analysis with the “glmnet” package (39). The candidate genes were consequently identified through the optimal penalty parameter *λ via* the 1 − SE (standard error) criterion. Finally, multivariate Cox analysis was used to determine the differentially expressed LRG targets for optimal LRPS construction based on a minimum Akaike information criterion (AIC) (40). Risk scores were computed by summing the expression of each differentially expressed LRG and the corresponding coefficient. Patients were divided into a low- and a high-risk group using the median risk scores. Furthermore, Kaplan–Meier analysis and time-dependent receiver operating characteristic (ROC) analysis were performed to assess the prognostic performance of the LRPS. PCA and *t*-distributed stochastic neighbor embedding (*t*-SNE) were utilized to investigate the distribution of the different subgroups. Data from E-MTAB-1980 and GSE22541 were used as two external independent validation cohorts to confirm the degree of generalization for the LRPS.

The relationships between the LRPS-based risk scores and the clinical characteristics (age, gender, tumor grade, and stage) were analyzed with the chi-square test in the TCGA and E-MTAB-1980 cohorts. In addition, we performed a stratified survival analysis for the patients classified into different subgroups to assess the robustness of the LRPS. Cox regression analyses were performed to determine the independent prognostic indicators. A nomogram combining the LRPS-based risk scores and clinical characteristics was developed using Cox regression coefficients predictive of the 1-, 3-, and 5-year OS in KIRC by employing the R packages “rms”, “regplot”, and “Hmisc”. The clinical availability nomogram was assessed using calibration curves, ROC curves, and decision curve analysis (DCA).

### Evaluation of the Immunogenomic Landscape

We exploited the TIMER (41), CIBERSORT (42), CIBERSORT-ABS, quanTIseq (43), MCP-counter (44), xCell (45), and EPIC (46) algorithms to investigate immune infiltration and function between the low- and high-risk groups based on the LRPS. Besides, we evaluated the abundance of 29 immune cells and immune-related molecules for each KIRC sample using the ESTIMATE algorithm. The differences in the immune score, stromal score, ESTIMATE score, and tumor purity were calculated and compared based on the gene expression profiles in the two groups.

### Evaluation of the Cancer–Immunity Cycle

The anticancer immune response, also called the cancer–immunity cycle, has seven steps in the TME. The activity score of each step was generated using Tracking Tumor Immunophenotype (TIP; http://biocc.hrbmu.edu.cn/TIP/index.jsp) (47). Then, we compared the differences in the activity scores of the seven steps to analyze the status of anticancer immunity and the proportion of tumor-infiltrating immune cells between different groups as in previous studies (48).

### Mutation and Evaluation of the Therapeutic Efficacy

To examine differences in the somatic mutations between the low- and high-risk groups, somatic mutations from TCGA were analyzed using the R package “maftools.” (49). Subsequently, we estimated the tumor mutational burden (TMB) of each patient between the two groups. To evaluate the therapeutic sensitivities between the two risk groups, we used computational methods to examine the effects of immunotherapy and chemotherapy. The immunophenoscore (IPS) was utilized to predict the immunotherapeutic responses (anti-PD-1 and anti-CTLA4) of the different groups based on the gene expression profiles using The Cancer Immunome Atlas (TCIA; https://tcia.at/) as described previously (38). For targeted therapeutic drug analysis, we adopted the “pRRophetic” package, which evaluates the half-maximal inhibitory concentration (IC_50_) through ridge regression based on the Genomics of Drug Sensitivity in Cancer (GDSC; https://www.cancerrxgene.org/) database (50). Moreover, we also analyzed the expression of the target genes of various drugs from the DrugBank (www.drugbank.ca) database.

### RNA Extraction and Quantitative Real-Time Polymerase Chain Reaction

A total of 30 pairs of fresh KIRC and adjacent non-tumor tissues were collected from Shandong Provincial Hospital (Jinan, Shandong) between 2020 and 2021. This study was approved by the Ethics Committee of Shandong Provincial Hospital, and informed consent was obtained from all enrolled patients. We validated the expression of genes using quantitative real-time PCR (qRT-PCR). In brief, total RNA isolation from the tissue was conducted with a TRIzol reagent (Invitrogen, Waltham, MA, USA). The quality and the concentration of total RNA were measured with a NanoDrop 2000 (Thermo Fisher Scientific, Waltham, MA, USA), then 500 ng RNA was used to synthesize cDNA using a reverse transcription kit (Takara, Shiga, Japan) following the manufacturer’s instruction. qRT-PCR analyses were applied using an Applied Biosystems 7500 Fast Real-Time PCR System (Thermo Fisher Scientific) with the SYBR Premix Ex Taq™ kit (Takara). GAPDH was used as an internal reference. The relative expression level was calculated with the 2^−ΔΔCT^ method. The primer sequences are listed in **Supplementary Table S1**.

### Statistical Analysis

All statistical analyses and graph visualization were implemented using R v4.1.1 (http://www.r-project.org). A *p* < 0.05 was considered to be significant, unless stated otherwise.

## Results

### Multi-Omics Landscape of LRGs in KIRC

**Figure 1:**
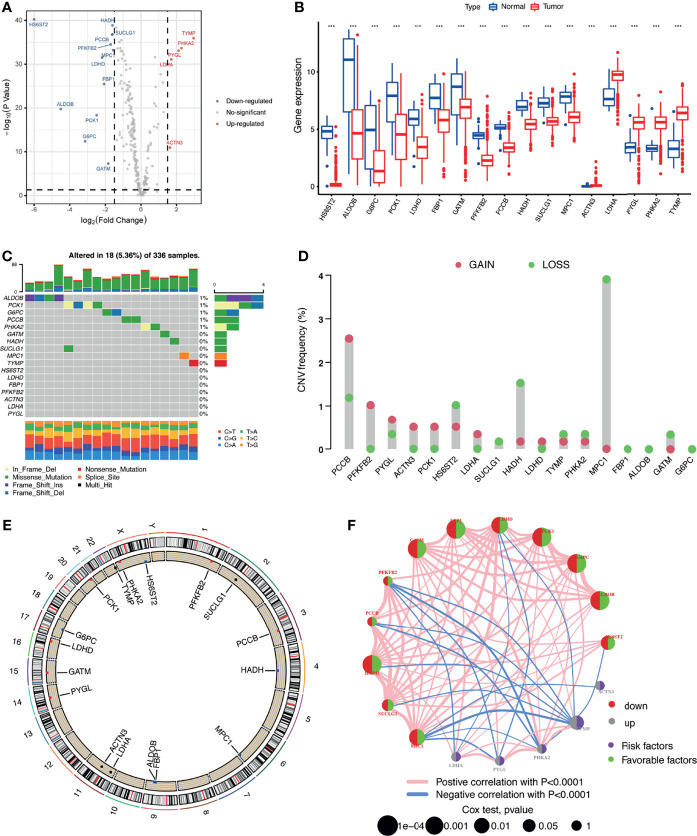
Multi-omics landscape of the differentially expressed lactate-related genes (LRGs) in kidney renal clear cell carcinoma (KIRC). **(A)** Volcano plot of the significantly dysregulated LRGs in KIRC tissue. **(B)** Boxplot of the expressions of the 17 differentially expressed LRGs in the TCGA-KIRC cohort. **(C)** Mutation frequency of the 17 differentially expressed LRGs in 336 patients with KIRC. **(D)** Copy number variations (CNVs) of the 17 differentially expressed LRGs. **(E)** Locations of the CNV alterations of the 17 differentially expressed LRGs on 23 chromosomes. **(F)** Correlations and prognosis of the 17 differentially expressed LRGs in patients with KIRC. ***p < 0.001.

Five priority LRG sets, including 267 genes, were selected from the MSigDB database. Based on the expression of these genes, we were able to discriminate KIRC tissues from normal controls by PCA, indicative of the different regulatory effects of lactate in normal kidney *vs.* KIRC tissues (**Supplementary Figure S1A**). We also observed that *TP53* appeared to be the most frequently mutated LRG in KIRC samples (**Supplementary Figure S1B**). To better clarify the impact of lactate on the progression of KIRC, we explored the mRNA expression profiles of the selected genes between the KIRC and normal samples. A total of 17 LRGs (*p* < 0.05 and |log_2_ FC| ≥ 1.50) were differentially expressed, among which 5 genes were upregulated and 12 were downregulated (**Figures 1A, B** **)**. We next assessed the incidence of mutation profiles and the copy number variations (CNVs) of the differentially expressed LRGs in KIRC. The mutations of these genes were similar—not more frequent. Among the 336 KIRC cases, only 18 (5.36%) had mutations in the five LRGs, including *ALDOB*, *PCK1*, *G6PC*, *PCCB*, and *PHKA2* (**Figure 1C**). We also found prevalent CNVs in most differentially expressed LRGs. Among them, *PCCB*, *PFKFB2*, and *PYGL* had comparatively high amplification, while *MPC1*, *HADH*, and *HS6ST2* showed primarily deletion in the investigation of CNV alterations (**Figure 1D**). The locations of CNV alterations of the 17 differentially expressed LRGs on chromosomes are shown in **Figure 1E**. The regulatory network depicted the comprehensive landscapes of the 17 LRGs concerning their interactions, correlation, and prognostic value (**Figure 1F**). We found that the upregulated genes presented a significant correlation with poor prognosis. Besides, close connections were established between the 17 LRGs using Spearman’s analyses (**Supplementary Figure S1C**).

### Identification of Lactate Clusters and Their Correlation with Biological Functions in KIRC

**Figure 2:**
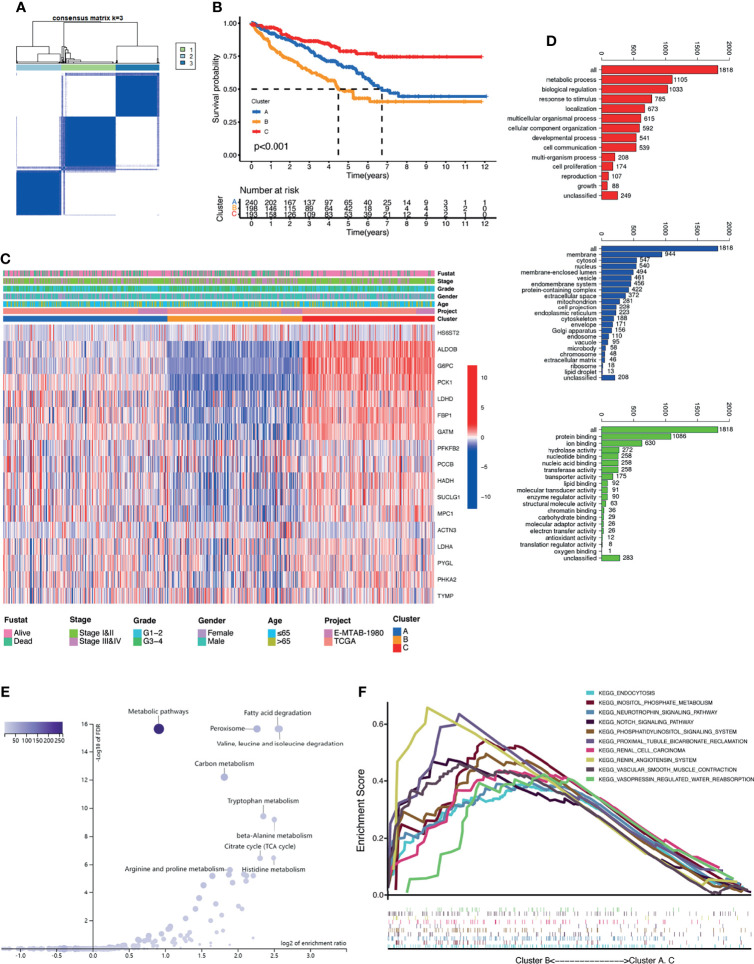
Clusters of differentially expressed lactate-related genes (LRGs) and biological characteristics in kidney renal clear cell carcinoma (KIRC). **(A)** Unsupervised clustering of the 17 differentially expressed LRGs and optimal consensus matrices for *k* = 3. **(B)** Survival analysis of three LRG clusters. **(C)** Unsupervised clustering of the 17 differentially expressed LRGs in the two KIRC cohorts (TCGA and E-MTAB-1980). **(D)** Gene Ontology (GO) annotation of 1,818 lactate phenotype-related differentially expressed genes (DEGs). **(E)** Kyoto Encyclopedia of Genes and Genomes (KEGG) pathway analysis of the above-mentioned genes. **(F)** Gene set enrichment analysis (GSEA) of several cancer-related pathways enriched in cluster B.

To further recognize the expression characteristics of lactate, we used an unsupervised clustering algorithm to classify the KIRC patients according to the expression of the 17 differentially expressed LRGs. The results showed that the optimal number of the clusters identified was three (*k* = 3), which was defined by the least crossover in the consensus matrix (**Figure 2A** and **Supplementary Figures S2A, B**), the smooth trend in the CDF (**Supplementary Figure S2C**), and no significant shift in the area under the curve (**Supplementary Figure S2D**). Accordingly, the entire cohort was sorted into clusters A (*n* = 240), B (*n* = 198), and C (*n* = 193). The results from PCA revealed distinct clustering of these three groups based on the expression of the 17 LRGs, indicating significant differences in the patterns of DEGs (**Supplementary Figure S2E**). Survival analysis revealed that the patients in cluster B suffered the worst prognosis, while cluster C patients had the best prognosis (**Figure 2B**). The expression profiles of the 17 LRGs and the clinical features in individual clusters were illustrated in a heatmap (**Figure 2C**). The three lactate clusters showed significant differences in the expression of LRGs, as expected, and most protective genes were upregulated in cluster C (**Supplementary Figure S2F**). Furthermore, three lactate clusters had surprisingly obvious distinct immune phenotypes. The abundance of the majority of antitumor immune cells, such as activated CD4 T cells and activated dendritic cells, was remarkably higher in cluster B than that in clusters A and C (**Supplementary Figure S2G**). Therefore, cluster B might be thought of as an immune-inflamed phenotype, while clusters A and C might be immune-desert phenotypes. Based on the above findings, we proposed that lactate serves an important role in the characteristics of immune infiltration.

To further investigate the heterogeneity of each lactate cluster, we identified 1,818 lactate phenotype-related DEGs (**Supplementary Figure S2H**) and then conducted functional enrichment analysis for these DEGs. GO analysis confirmed that these DEGs were concentrated on biological processes related to metabolic processes (**Figure 2D**). KEGG pathway analysis showed enrichment of metabolic pathways and peroxisomes (**Figure 2E**). GSEA suggested that cluster B was closely related to several cancer-related pathways (**Figure 2F**), which may explain the inconsistent clinical outcomes among diverse lactate clusters. Taken together, it is reasonable to suggest that lactate might have a non-negligible role in the development of KIRC.

### Construction of LRPS in TCGA Database

**Figure 3:**
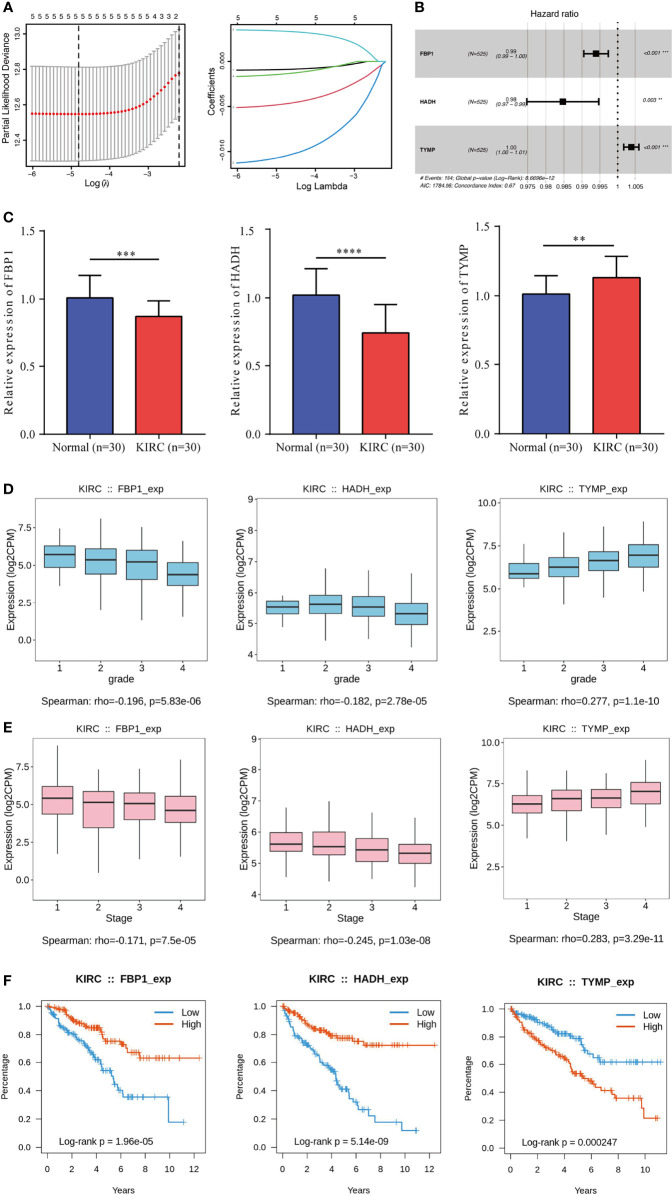
Construction of a lactate-related prognostic signature (LRPS) in The Cancer Genome Atlas (TCGA) cohort. **(A)** Coefficient profiles of the six lactate-related genes (LRGs) and identification of the best parameter (*lambda*) in the LASSO (least absolute shrinkage and selection operator). **(B)** Three genes retained to construct the optimal LRPS using multivariate Cox analysis. **(C)** mRNA levels of three LRGs detected by qRT-PCR. **(D, E)** Correlations of the three LRGs in LRPS and tumor grade **(D)** and tumor stage **(E)**. **(F)** Kaplan–Meier survival analysis of the three LRGs in LRPS. **p < 0.01, ***p < 0.001, ****p < 0.0001.

To construct an LRPS for prognosis indication of patients with KIRC, we conducted univariate Cox regression on the 17 differentially expressed LRGs and selected six genes that were significantly linked with OS using TCGA (**Table 2**). To eliminate the effect of overfitting, LASSO analysis was employed and five genes were retained (**Figure 3A**). Subsequently, three genes, namely, two protective genes (*FPB1* and *HDAH*) and one risk gene (*TYMP*), were retained to construct the optimal LRPS based on the minimum AIC value by multivariate Cox regression (**Figure 3B**). Then, we examined the mRNA levels of the three targets with qRT-PCR and found that *FPB1* and *HDAH* had lower expressions, while *TYMP* had a higher expression in KIRC samples than in adjacent non-tumor samples (**Figure 3C**). Using the Human Protein Atlas (HPA) database (https://www.proteinatlas.org/), we also found a consistent trend in the protein levels of these genes (**Supplementary Figure S3**). The TISIDB (http://cis.hku.hk/TISIDB/index.php) database was further utilized to study the prognostic value of the three genes and their correlation with the clinicopathological characteristics. We noticed that the mRNA expressions of *FPB1* and *HDAH* were negatively correlated with tumor grade in patients with KIRC, while that of *TYMP* was positively correlated (**Figure 3D**). The same pattern was observed in the tumor stage (**Figure 3E**). Kaplan–Meier analyses confirmed that patients with low expressions of *FPB1* and *HDAH* and a high expression of *TYMP* suffered poor prognosis (**Figure 3F**).

**Table 2:** Univariate Cox regression analysis of the 17 differentially expressed lactate-related genes.

Genes	HR	UI of 95%CI	LI of 95%CI	*p*-value
*HADH*	0.9763	0.9672	0.9855	5.35E−07
*FBP1*	0.9925	0.9893	0.9957	3.86E−06
*TYMP*	1.0048	1.0026	1.0070	1.41E−05
*GATM*	0.9967	0.9951	0.9984	8.17E−05
*MPC1*	0.9912	0.9862	0.9963	0.0006
*LDHD*	0.9751	0.9608	0.9896	0.0008
*PCK1*	0.9973	0.9953	0.9994	0.0110
*HS6ST2*	1.0606	1.0131	1.1104	0.0119
*SUCLG1*	0.9925	0.9853	0.9997	0.0409
*LDHA*	0.9995	0.9991	1.0000	0.0419
*G6PC*	0.9879	0.9763	0.9997	0.0442
*PHKA2*	0.9961	0.9901	1.0021	0.2046
*PCCB*	0.9885	0.9660	1.0115	0.3236
*ALDOB*	1.0001	0.9998	1.0004	0.5047
*ACTN3*	1.1375	0.6641	1.9484	0.6389
*PFKFB2*	0.9971	0.9824	1.0121	0.7052
*PYGL*	0.9992	0.9932	1.0053	0.8000

HR, hazard ratio; CI, confidence interval; UI, upper limit; LI, lower limit.

**Figure 4:**
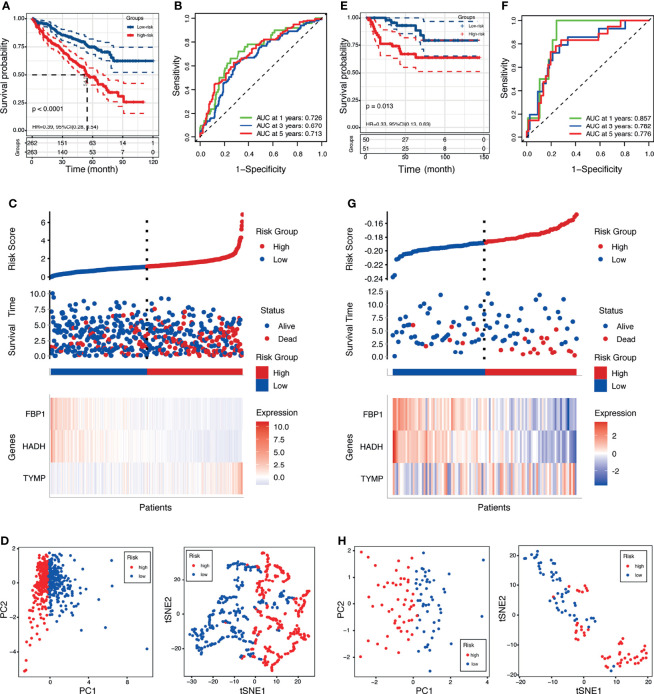
Prognostic value of the lactate-related prognostic signature (LRPS) in patients with kidney renal clear cell carcinoma (KIRC). **(A, E)** Kaplan–Meier analysis of overall survival (OS) in The Cancer Genome Atlas (TCGA) and E-MTAB-1980 cohorts. **(B, F)** Receiver operating characteristic (ROC) analysis of the LRPS in predicting the 1-, 3-, and 5-year OS in the TCGA and E-MTAB-1980 cohorts. **(C, G)** Distribution plots of the risk score, OS status, and heatmap of gene expressions in the TCGA and E-MTAB-1980 cohorts. **(D, H)** Principal component analysis (PCA) and *t*-distributed stochastic neighbor embedding (*t*-SNE) plot of the TCGA and E-MTAB-1980 cohorts.

The risk scores were computed with a formula that included the expression of the three genes and their coefficients: risk score = *FPB1* × (−0.006) + *HDAH* × (−0.015) + *TYMP* × 0.004. Using the median risk score, the patients were assigned into a low-risk group and a high-risk group. In TCGA, survival was markedly longer in the low-risk group than in the high-risk group (**Figure 4A**). The area under the ROC curve (AUC) values were 0.726, 0.670, and 0.713 for 1-, 3-, and 5-year survival, respectively (**Figure 4B**). The risk score plot and living status indicated that the low-risk group had better survival status and longer survival time (**Figure 4C**). In addition, the heatmap of the expressions of the three genes showed that the two protective genes (*FPB1* and *HDAH*) were downregulated and the one risk gene (*TYMP*) was upregulated in the high-risk subgroup. The samples in the two subgroups were further divided into two distribution patterns using PCA and *t*-SNE (**Figure 4D**). The results revealed that the LRPS might effectively predict the outcomes of patients with KIRC.

### Validation of LRPS in Two Independent External Cohorts

To evaluate the universality of the constructed LRPS from the training cohort, two independent cohorts (E-MTAB-1980 and GSE22541) were introduced as validation groups. The risk score of each patient in these two validation cohorts was computed using the same formula as that for the TCGA cohort. For the E-MTAB-1980 cohort, patients in the high-risk group suffered poorer survival status than those in the low-risk group (**Figure 4E**). In addition, the AUC values of the LRPS according to ROC analysis were 0.857 in 1 year, 0.782 in 2 years, and 0.776 in 3 years (**Figure 4F**). The distribution plot of the risk score, survival status, and expressions of the three genes showed that there was an increase in mortality with an increasing risk score (**Figure 4G**). PCA and *t*-SNE illustrated the diverse direction distribution in the two risk subgroups (**Figure 4H**). The same analyses were conducted in the GSE22541 cohort, and similar findings were obtained (**Supplementary Figure S4**).

### Analysis of the Correlation Between LRPS and Clinical Features

**Figure 5:**
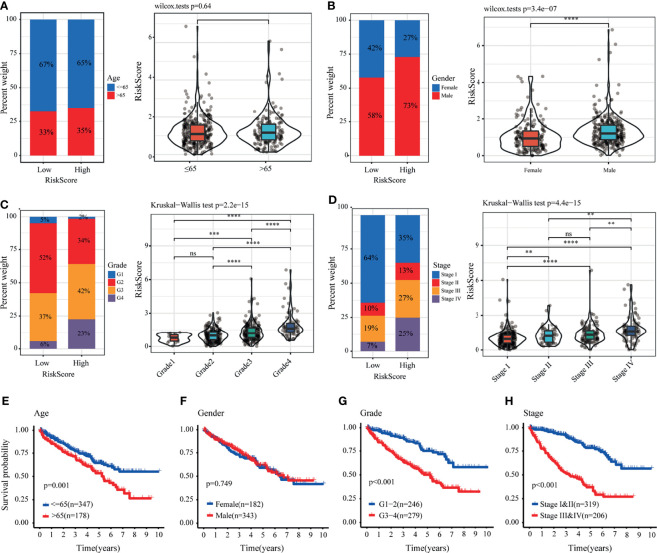
Correlation between the lactate-related prognostic signature (LRPS) and clinical features in The Cancer Genome Atlas (TCGA) cohort. **(A–D)** Proportion of clinical features (age, gender, tumor grade, and stage) in the low- or high-risk group. Distribution of risk scores in different groups according to clinical features. **(E–H)** Kaplan–Meier analysis for patients with kidney renal clear cell carcinoma (KIRC) based on the LRPS stratified by clinical features (age, gender, tumor grade, and stage). **p < 0.01, ***p < 0.001, ****p < 0.0001. *ns*, not significant.

We further analyzed the value of the LRPS between the two groups stratified by different clinical factors in TCGA. Male patients with advanced tumor grade and stage had higher risk scores, but no significant difference was found for age (**Figures 5A** **–D**). Stratified survival analysis indicated that patients who were older (**Figure 5E**) and with advanced tumor grade (**Figure 5G**) and TNM stage (**Figure 5H**) tended to have poorer OS, while the gender-specific survival analysis did not show differences (**Figure 5F**). In addition, we also found that patients in the high-risk group were closely linked to a poorer outcome across all subgroups, except for those with tumor grades 1–2 (**Supplementary Figures S5A–D**). In the E-MTAB-1980 cohort, men were at higher risk than women, whereas the risk scores of patients with advanced tumors were higher relative to those with early tumor grade and TNM stage (**Supplementary Figures S6B–D**). No differences were noticed between patients stratified by age (**Supplementary Figure S6A**). In addition, Kaplan–Meier analyses based on the tumor grade and TNM stage with significant OS differed between the two groups (**Supplementary Figures S6E–H**). There were significant differences among the four subgroups, including older age (≤65 years), being men, with tumor grades 3–4, and with tumor stages III–IV (**Supplementary Figures S5E–H**).

To investigate the independence of LRPS and other clinical factors (age, gender, tumor grade, and TNM stage), both univariate and multivariate Cox regression analyses were conducted (**Supplementary Table S2**). In the TCGA cohort, univariate Cox regression revealed that age, grade, and stage were associated with the OS of patients with KIRC. The stage and risk score remained significant using multivariate Cox regression analysis. Thus, the stage and risk score were regarded as independent prognostic indicators, which were confirmed in the E-MTAB-1980 dataset.

### Development of a Clinical Nomogram

**Figure 6:**
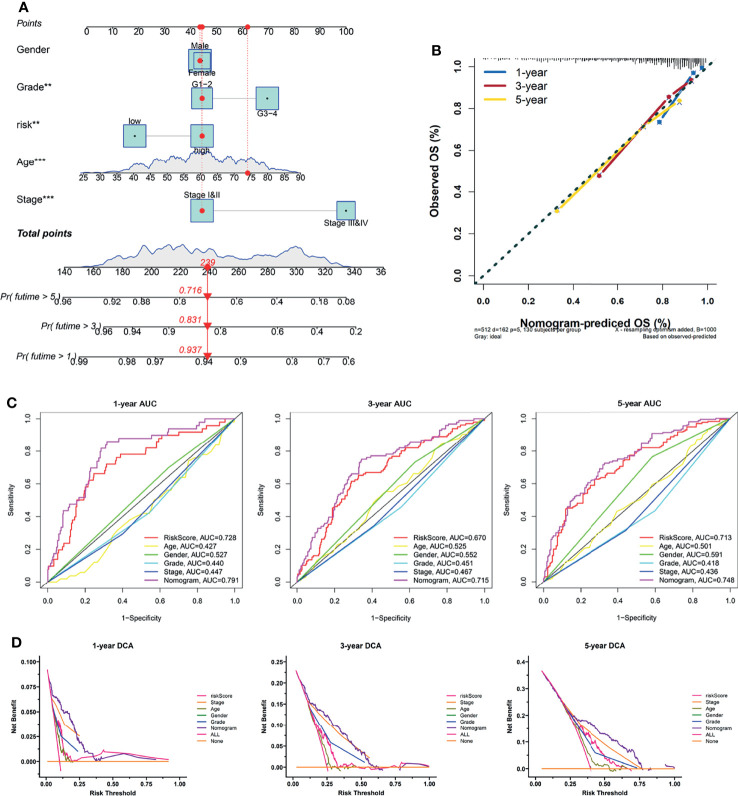
Construction of a nomogram predicting the overall survival (OS) of patients with kidney renal clear cell carcinoma (KIRC) in The Cancer Genome Atlas (TCGA) cohort. **(A)** Nomogram based on the lactate-related prognostic signature (LRPS), age, gender, tumor grade, and stage. **(B)** Calibration curves for internal validation of the nomogram. **(C)** Time-dependent receiver operating characteristic (ROC) curves of the nomogram in predicting the 1-, 3-, and 5-year OS. **(D)** Decision curve analysis (DCA) of the nomogram. **p < 0.01, ***p < 0.001.

Subsequently, we developed a nomogram for OS prediction using clinical parameters and the LRPS-based risk scores in the TCGA (**Figure 6A**) and E-MTAB-1980 (**Supplementary Figure S7A**) cohorts. A calibration plot for internal validation of the nomogram showed excellent consistency between the nomogram-predicted probability and actual observations of the 1-, 3-, and 5-year OS (data for the TCGA and E-MTAB-1980 cohorts are shown in **Figure 6B** and **Supplementary Figure S7B**, respectively). In the TCGA cohort, the AUCs of the combined nomogram for 1-, 3-, and 5-year OS were 0.728, 0.715, and 0.748, respectively, increasing the efficiency of the other clinical factors for predicting OS (**Figure 6C**). Additionally, the AUCs of the nomogram at 1, 3, and 5 years (0.936, 0.922, and 0.898, respectively) showed satisfactory accuracy in the E-MTAB-1980 cohort (**Supplementary Figure S7C**). The DCA curves exhibited that the nomogram obtained a higher net benefit in both cohorts (TCGA and E-MTAB-1980 cohorts in **Figure 6D** and **Supplementary Figure S7D**, respectively). These results indicated that the predictive nomogram for OS performed with improved accuracy and could help clinical management.

### Immune Landscape of LRPS Groups

**Figure 7:**
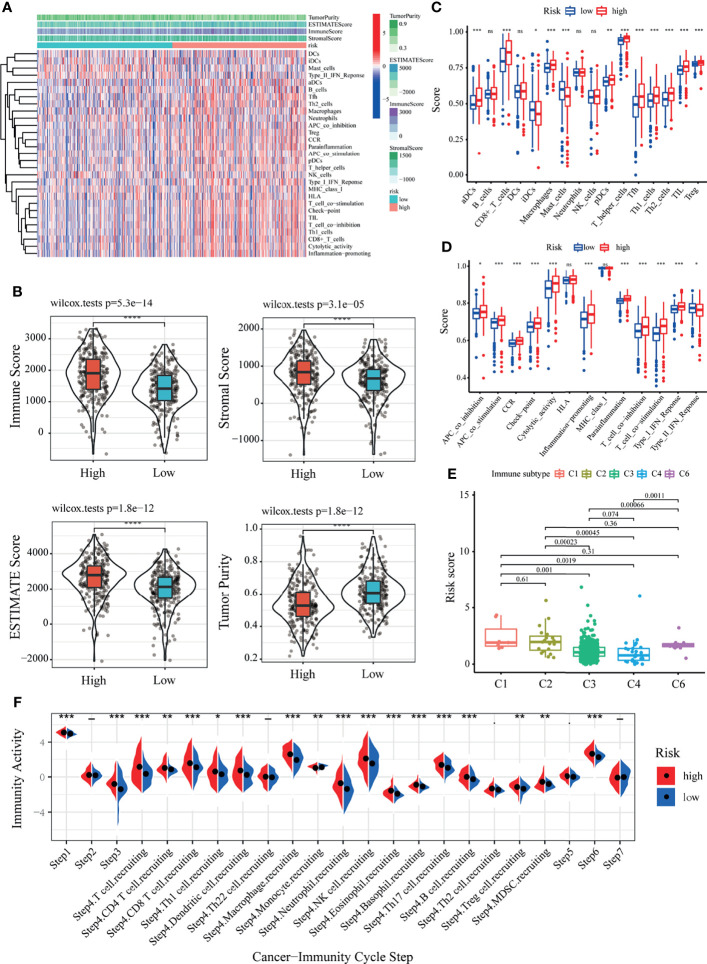
Correlation of the lactate-related prognostic signature (LRPS) with the immune landscape of patients with kidney renal clear cell carcinoma (KIRC). **(A)** Enrichment levels of immune-related cells and types between the low- and high-risk groups. **(B)** Violin plot showing the differences in the immune score, stromal score, ESTIMATE score, and tumor purity between the low- and high-risk groups. **(C)** Correlation between the risk score and immune subtypes. **(D)** Differences in the 16 immune cells between the low- and high-risk groups. **(E)** Differences in the 13 immune-related functions between the low- and high-risk groups. **(F)** The difference of the abundance of antitumor immune cells between low- and high-risk groups. *p < 0.05, **p < 0.01, ***p < 0.001, ****p < 0.0001. *ns*, not significant.

To further understand the underlying correlation of the risk score with the immune landscape of the KIRC samples, the differences in the various immune cell components between the low- and high-risk groups were compared. According to seven algorithms, a heatmap of the various immune cell components is shown in **Supplementary Figure S8A**. The correlation coefficients of the components with LRPS-based risk scores were calculated using Spearman’s analysis and visualized in a lollipop plot (**Supplementary Figure S8B**). Subsequently, the immune score, stromal score, ESTIMATE score, and tumor purity were calculated for the low- and high-risk groups using the ESTIMATE algorithm (**Figure 7A**). The high-risk group demonstrated a higher immune score, stromal score, ESTIMATE score, but a lower tumor purity than the low-risk group (*p* < 0.05) (**Figure 7B**), implying that several immune cells and immune-related molecules were abundant in the high-risk group. Various tumor-infiltrating immune cells were positively correlated with a higher risk score, indicating the significant impacts of these cells on the pathogenesis of KIRC (**Figure 7C**). Moreover, the scores of all immune-related molecules were significantly higher in the high-risk group, including that of human leukocyte antigen (HLA), major histocompatibility complex (MHC) class I, and type II interferon (IFN) response (**Figure 7D**).

To further confirm the reliability of the LRPS on immunotyping, we next exploited the correlation between the risk scores and the previously reported pan-cancer immune subtypes. In the present study, KIRC patients with the C1, C2, and C6 subtypes showed higher risk scores compared to those with the other two immune subtypes (**Figure 7E**). In contrast, the risk scores were significantly lower for patients with the C3 and C4 subtypes. It is well known that C3 and C6 are related to better and inferior outcomes, respectively. These results demonstrated the unique characteristics of the KIRC immune microenvironment, offering a conducive complement to previous studies. An antitumor immune response must launch a sequence of stepwise events to eliminate cancer cells effectively (51). To further explore the impact of immune cells on KIRC, we calculated the immune activity score of each step using TIP analysis with the RNA expression data. We then examined the differences in the scores of the seven steps among two groups. The results showed that the abundance of antitumor immune cells was higher in the high-risk group than that in the low-risk group (**Figure 7F**).

### Mutation and Immunotherapeutic Responses of LRPS Groups

**Figure 8:**
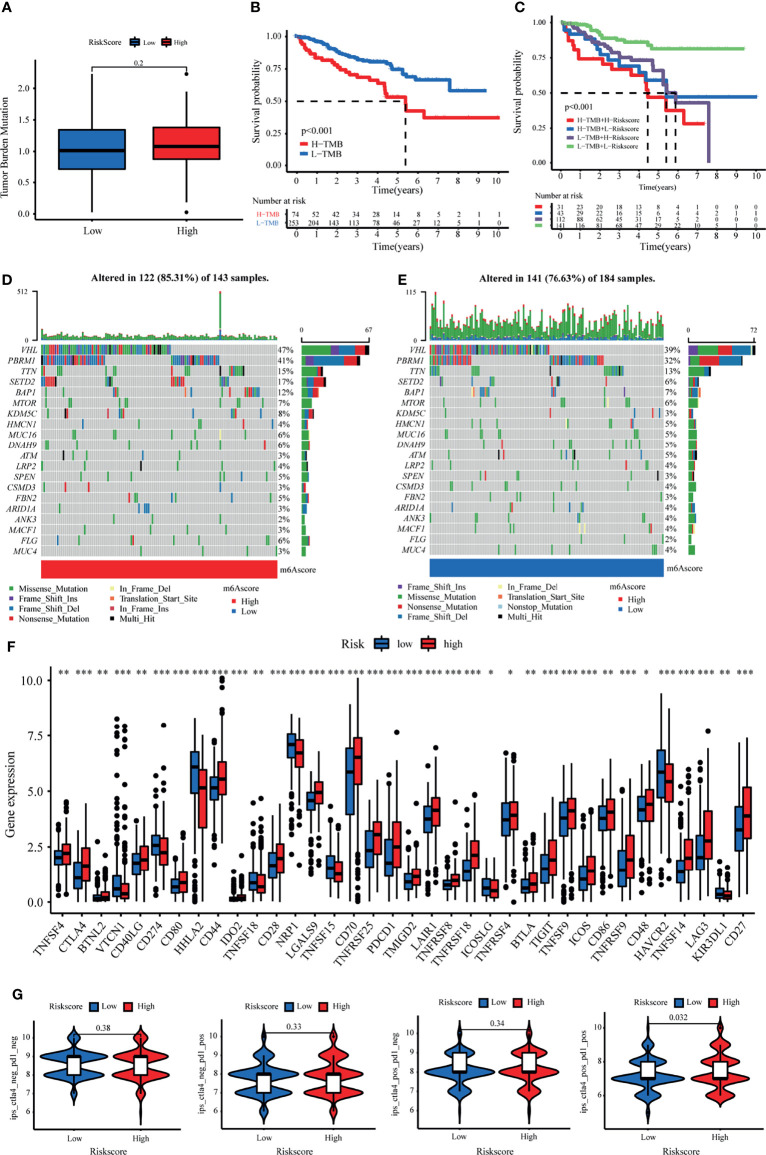
Tumor mutational burden (TMB) and immunotherapeutic responses of the lactate-related prognostic signature (LRPS). **(A)** TMB between the low- and high-risk subgroups based on LRPS. **(B)** Survival analysis of the different groups stratified by TMB. **(C)** Survival analysis of distinct groups stratified by both TMB and LRPS. **(D, E)** Waterfall plot of tumor somatic mutation in the high- and low-risk groups. **(F)** Differences in the expressions of immune checkpoints between the low- and high-risk groups. **(G)** Comparison of the immunophenoscore (IPS) between the low- and high-risk groups stratified by both CTLA4 and PD-1. *p < 0.05, **p < 0.01, ***p < 0.001.

Considering that the TMB is closely related to the efficacy of immunotherapy, we estimated the value of TMB between the two risk groups based on LRPS. As expected, the high-risk subgroup possessed a higher TMB through TMB quantification analysis (**Figure 8A**). Consequently, patients with low TMB demonstrated a satisfactory survival benefit (**Figure 8B**). We then explored the value of combining the risk score and TMB in predicting the outcomes of patients. Results of the Kaplan–Meier analysis suggested that a low risk score and a low TMB are linked with a longer survival (**Figure 8C**). Subsequently, we further investigated the distribution patterns of the top 20 somatic mutations between the two groups based on TCGA using the “maftools” package. The most common mutations were *VHL* and *PBRM1*, with a rate of the 20th most significant mutated gene of 85.31% *versus* 76.63% (**Figures 8D, E** **)**. Accumulated evidence demonstrated that a high TMB was linked with a better outcome of immunotherapy. Considering the importance of checkpoint inhibitors in clinical treatment, we further analyzed the differences in the expressions of ICBs and found substantial differences in *PD-*1, *CTLA4*, *LAG3*, and *CD27* between the two groups (**Figure 8F**). Moreover, we specifically investigated the significance of the risk scores to assess the effect of immunotherapy using TCIA. The results illustrated that the relative probabilities of responding to CTLA4_positive_/PD-L1_positive_ treatments in the high-risk group were higher than those in the low-risk group (**Figure 8G**). This suggested that patients in the high-risk group might be more likely to respond to CTLA4_positive_/PD-L1_positive_ immunotherapy and thus obtain more satisfactory clinical outcomes.

### Drug Susceptibility Analysis of LRPS Groups

**Figure 9:**
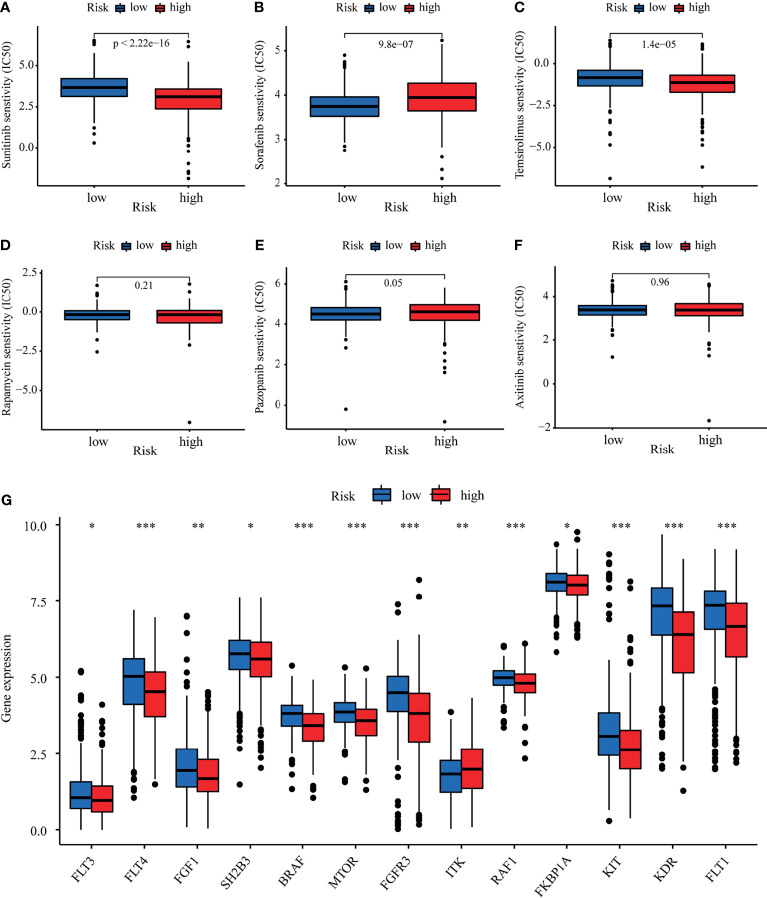
Evaluation of therapeutic response by the lactate-related prognostic signature (LRPS). **(A–F)** Sensitivity analysis for sunitinib **(A)**, sorafenib **(B)**, temsirolimus **(C)**, rapamycin **(D)**, pazopanib **(E)**, and axitinib **(F)** in patients at low and high risk. **(G)** Differences in the expressions of the target genes after targeted drug therapy between the low- and high-risk groups. *p < 0.05, **p < 0.01, ***p < 0.001.

We explored the association between the LRPS-based risk scores of patients with KIRC and their response to six common anticancer drug agents (sunitinib, temsirolimus, sorafenib, pazopanib, rapamycin, and axitinib) using the pRRophetic algorithm. We calculated the IC_50_ of these agents in the low- and high-risk groups and observed that patients in the low-risk group were significantly more sensitive to sunitinib and temsirolimus, while sorafenib and pazopanib had higher IC_50_ values in the high-risk group (**Figures 9A** **–F**). We further evaluated the expressions of the target genes—based on targeted drug therapy—in advanced KIRC in the two groups. These target genes from the DrugBank database included *FLT1*, *FLT3*, *FLT4*, *FGF1*, *FGFR3*, *SH2B3*, *BRAF*, *MTOR*, *ITK*, *RAF1*, *FKBP1A*, *KIT*, and *KDR* (**Supplementary Table S3**). Interestingly, all of the target genes exhibited significant differences in their expressions between the two groups (**Figure 9G**). These findings suggested that the risk score might distinguish more suitable patients to receive appropriate therapy.

### Pan-Cancer Analysis of *TYMP*

To study the functional implication of the risk gene (*TYMP*) in cancer development, we performed a pan-cancer analysis. Among the 20 cancer types in TCGA, the expression of *TYMP* was elevated in 75% (15/20) of tumor tissues relative to normal controls (**Supplementary Figure S9A**). We investigated the links between the tumor-infiltrating lymphocyte (TIL) markers and *TYMP*. The results suggested that the expression of *TYMP* was notably related to the expression of immunocyte markers and potentially involved in regulating the immune response (**Supplementary Figure S9B**). In addition, we evaluated the associations between the expression of *TYMP* and the levels of TMB and noticed that the mRNA level of *TYMP* was linked to several cancers, including KIRC (**Supplementary Figure S9C**). Microsatellite instability (MSI) is a unique molecular alteration caused by defects in DNA mismatch repair and is potentially involved in the increased immunogenicity of tumor cells. The radar plot displayed the association of *TYMP* expression with MSI in several cancers, including KIRC (**Supplementary Figure S9D**). Besides, the expression of *TYMP* was correlated with the expressions of the DNA mismatch repair (MMR) genes (*MLH1*, *MSH2*, *MSH6*, *PMS2*, and *EPCAM*) and DNA methylation regulatory genes (*DNMT1*, *DNMT2*, *DNMT3A*, and *DNMT3B*) in various cancer types (**Supplementary Figures S9E, F**).

## Discussion

KIRC is the primary subtype of RCC with high heterogeneity and metastatic potential and immune-responsive tumors. Despite the increasing incidence of KIRC, the survival of patients with advanced RCC has been markedly improved with molecular target–drug combinations (sunitinib and pazopanib) combined and ICB (nivolumab). However, the complexity of the TME in RCC, including the accumulation of lactate, results in insufficient therapeutic response and resistance to drugs, as well as relapse during treatment in some patients. Therefore, it is imperative to comprehensively investigate LRGs to predict the outcomes and therapeutic responses of new treatment targets for patients with KIRC. To the best of our knowledge, this is the first research exploring risk signatures for the prediction of prognosis and therapeutic efficacy in KIRC.

In our study, we identified 17 differentially expressed LRGs, three of which were determined to construct an LRPS using LASSO and Cox regression analyses. The signature could classify patients with KIRC into low- and high-risk groups. The performance of this signature was confirmed in two independent validation cohorts, demonstrating its robust survival prediction efficiency for KIRC. Moreover, the results suggested that LRPS can serve as an independent prognostic factor. Simultaneously, ROC analysis was applied to illustrate its assessment of time-associated outcomes in patients, which indicated a relatively high diagnostic performance in predicting short-term survival (1-year OS, AUC = 0.726) compared to long-term survival (5-yr OS, AUC = 0.713) in the TCGA cohort. Accordingly, we also found similar results for the external validation cohorts. An explanation might be that the complicated mechanisms of KIRC are influenced by various factors, not just the LRGs that contributed to tumor progression; thus, some other important genes should also be incorporated into the signature in the future. In addition, the LRPS-based risk scores presented a significant positive correlation with TNM stage (stages I/II *vs.* stages III/IV) in the TCGA cohort, which was consistent with the results in the E-MTAB-1980 cohort, confirming the prognostic merit of our signature. Moreover, a nomogram was constructed *via* the integration of the LRPS-based risk scores with clinical factors (age, gender, tumor stage, and grade), which could guide the follow-up of individual treatments.

Three genes [fructose-1,6-bisphosphatase 1 (*FBP1*), short-chain-*L*-3-hydroxyacyl-CoA dehydrogenase (*HADH*), and thymidine phosphorylase (*TYMP*)] included in the constructed signature have previously been associated with the progression of multiple cancers. The gluconeogenic rate-limiting enzyme FBP1, which resided on chromosome 9q22, was found to inhibit tumor growth in several cancer types, among others also in KIRC (52). Li et al. revealed that FBP1 could regulate the uptake of glucose and the secretion of lactate by alleviating the level of glycolysis and NADPH in KIRC cells under the influence of hypoxia-inducible factors (HIFs). Furthermore, FBP1 suppressed HIF activity in the nucleus through direct interaction with a HIFα inhibitory domain, independent of its enzymatic activity (53). The *HADH* gene, which consists of 10 exons, encodes HADH, which is a key enzyme in the third step of the fatty acid β-oxidation (54). Recently, growing evidence has demonstrated its significant role in different carcinomas. For example, several studies have demonstrated that the overexpression of *HADH* was related to poor clinical outcomes in acute myeloid leukemia and colon cancer (55, 56). Additionally, Shen et al. illustrated that the downregulation of *HADH* facilitated gastric cancer cell migration and invasion through activating the Akt signaling pathway, associated with more advanced stage and poorer outcomes (57). In patients with KIRC, a decreased expression of *HADH* was also related to immune infiltration and poor prognosis (58). TYMP, also known as a platelet-derived endothelial cell growth factor, catalyzes the reversible phosphorolysis of thymidine (59). *TYMP* was shown to be upregulated in multiple solid tumors, where it is implicated in cell proliferation and angiogenesis (60). It was previously demonstrated that both tumor cells and the surrounding matrix cells expressed *TYMP* in the TME (60). Our results suggested that the expression of *TYMP* was notably related to the expression of several immunocyte markers and might be involved in regulating the immune response. Many studies have indicated that *TYMP* was a potential target for cancer immunotherapy (59, 61). Our study suggested that the prognostic value of these three LRGs in KIRC and the mechanism of the three genes in KIRC need further investigation.

The TME consists of tumor cells, various infiltrating immune cells, stromal cells, and cytokines. Among these components, infiltrating immune cells play a significant role in tumor growth, invasion, metastasis, and in modulating anticancer immunity. Therefore, it serves as a promising therapeutic target (62). In the present study, we first divided the patients into three clusters with distinct immune features using unsupervised clustering based on the expressions of 17 differentially expressed LRGs. Patients in cluster B, with a higher proportion of activated CD4, CD8 T cells, natural killer cells, macrophages, and some regulatory T cell infiltration, presented with an improved prognosis than those in clusters A and C. Intriguingly, we also found different immune components between the different risk groups classified based on the LRPS-based risk scores. Both targeted therapy and ICBs, as first-line treatments, play an irreplaceable role in the treatment of KIRC (63). However, there are still difficulties in determining the optimal treatment for individuals. CTLA4 and PD-1 are both critical ICBs. By comparing the expressions of the immune checkpoint genes (*CTLA4* and *PD-1*) stratified by the risk score, we found a significant difference between the two risk groups in patients receiving positive CTLA4 and PD-L1 treatments. Moreover, patients with different risk scores might exhibit distinct sensitivity treatments with sunitinib, temsirolimus, and sorafenib. In line with the different sensitivities to targeted drugs, the target genes of these drugs, such as *FLTs*, *KIT*, and *mTOR*, exhibited significant differences between the two groups. These findings implied that the LRPS-based risk score may be a remarkable marker for assessing the response to targeted therapy and immunotherapy to facilitate the development of personalized therapy for KIRC.

Despite the merits of our findings, several limitations should be noted. Firstly, the findings were constructed and validated retrospectively in public databases. Therefore, prospective research is essential to evaluate the clinical utility of the signature in patients with KIRC. Moreover, comprehensive functional experiments are essential to illuminate the elusive mechanisms of the three lactate-related genes.

To summarize, we first identified the differentially expressed LRGs in KIRC and divided KIRC patients into three clusters. These clusters presented significant differences in prognosis and immune cell infiltration. Furthermore, we constructed a novel prognostic signature using three LRGs (*FBP1*, *HADH*, and *TYMP*) in patients with KIRC, which can serve as a robust predictor of prognosis and response to immunotherapy. In conclusion, the constructed LRPS can provide important insights for subsequent mechanisms in functional research and guide clinicians in making rational treatment decisions.

## Data Availability Statement

Publicly available datasets were analyzed in this study. This data can be found here: https://portal.gdc.cancer.gov/, https://www.ncbi.nlm.nih.gov/, and https://www.ebi.ac.uk/.

## Ethics Statement

The studies involving human participants were reviewed and approved by Shandong Provincial Hospital. The patients/participants provided their written informed consent to participate in this study.

## Author Contributions

ZS: designed the study and wrote original draft. WT: collected data and wrote the manuscript. XG: analyzed and interpreted the data. CJ: participated in the revision and language editing. MZ and ZW: prepared tables and figures. FK and NS: helped with the manuscript and data review. SJ and HW: edited and revised the manuscript. All authors have seen and approved the final version of the manuscript.

## Funding

The research was supported by the Shandong Key Research and Development Program, China (2019GSF108263).

## Conflict of Interest

The authors declare that the research was conducted in the absence of any commercial or financial relationships that could be construed as a potential conflict of interest.

## Publisher’s Note

All claims expressed in this article are solely those of the authors and do not necessarily represent those of their affiliated organizations, or those of the publisher, the editors and the reviewers. Any product that may be evaluated in this article, or claim that may be made by its manufacturer, is not guaranteed or endorsed by the publisher.
