# Supplementary material for: Construction of a Lactate-Related Prognostic Signature for Predicting Prognosis, Tumor Microenvironment, and Immune Response in Kidney Renal Clear Cell Carcinoma
Source: Front Immunol. 2022 Feb 17;13:818984. doi: 10.3389/fimmu.2022.818984 (PMC8892380; doi:10.3389/fimmu.2022.818984)
Supplement: Supplementary file 1 [file DataSheet_1.docx]

**Supplementary Materials**

**Supplementary Tables**

| Genes | Forward Primer | Reverse Primer |
| --- | --- | --- |
| FBP1 | 5′-ATCGGGGAGTTCATTTTGGT-3′ | 5′-CATTGGGGCTCTTCTTGTTA-3′ |
| HADH | 5′-GCTAATGCCACCACCAGACAA-3′ | 5′-CGTCACCTCGTTCATACAGCC-3′ |
| TYMP | 5'-CCTGCGGACGGAATCCT-3' | 5'- GCTGTGATGAGTGGCAGGCT -3' |
| β-actin | 5′-TTAGTTGCGTTACACCCTTTC-3′ | 5′-ACCTTCACCGTTCCAGTTT-3′ |

Table S1. The primers for qRT-PCR.

Table S2. Univariate and multivariate Cox regression analysis of clinical factors and prognostic risk signature in the TCGA cohort and the E-MTAB-1980 cohort.

| **Variable** | **TCGA cohort** | | | | **E-MTAB cohort** | | | | |
| --- | --- | --- | --- | --- | --- | --- | --- | --- | --- |
|  | **Univariate** | | **Multivariate** | | **Univariate** | | | **Multivariate** | |
|  | HR | *P* | HR | *P* | HR | *P* | | HR | *P* |
| **Age**  ≤65 vs >65 | 1.029 | <0.001 | 1.032 | <0.001 | 1.045 | | 0.035 | 1.034 | 0.141 |
| **Gender**  Female vs Male | 0.957 | 0.789 | 0.954 | 0.779 | 2.366 | | 0.164 | 2.448 | 0.175 |
| **Grade**  G1-2 vs G3-4 | 2.326 | <0.001 | 1.362 | 0.013 | 3.024 | | <0.001 | 1.411 | 0.342 |
| **Stage**  Ⅰ/Ⅱ vs Ⅲ/Ⅳ | 1.939 | <0.001 | 1.663 | <0.001 | 2.307 | | <0.001 | 2.185 | <0.001 |
| **Risk score**  Low vs High | 1.793 | <0.001 | 1.366 | <0.001 | 16.366 | | <0.001 | 18.543 | <0.05 |

Table S3. The targets genes of six anticancer drugs agents from the DrugBank dataset.

| **Drugs**  **No.** | **Sunitinib** | **Sorafenib** | **Temsirolimus** | **Pazopanib** | **Axitinib** | **Rapamycin** |
| --- | --- | --- | --- | --- | --- | --- |
| 1 | VEGFR2 | VEGFR2 | MTOR | VEGFR2 | VEGFR2 | MTOR |
| 2 | KIT | KIT |  | KIT | FLT1 | FGF2 |
| 3 | PDGFRB | PDGFRB |  | PDGFRB | FLT4 | FKBP1A |
| 4 | FLT3 | FLT3 |  | FLT1 |  |  |
| 5 | FLT1 | FLT1 |  | FLT4 |  |  |
| 6 | FLT4 | FLT4 |  | PDGFRA |  |  |
| 7 | CSF1R | RAF1 |  | FGFR3 |  |  |
| 8 | PDGFRA | BRAF |  | FGF1 |  |  |
| 9 |  | FGFR1 |  | SH2B3 |  |  |
| 10 |  | RET |  | ITK |  |  |

**Supplementary Figures**


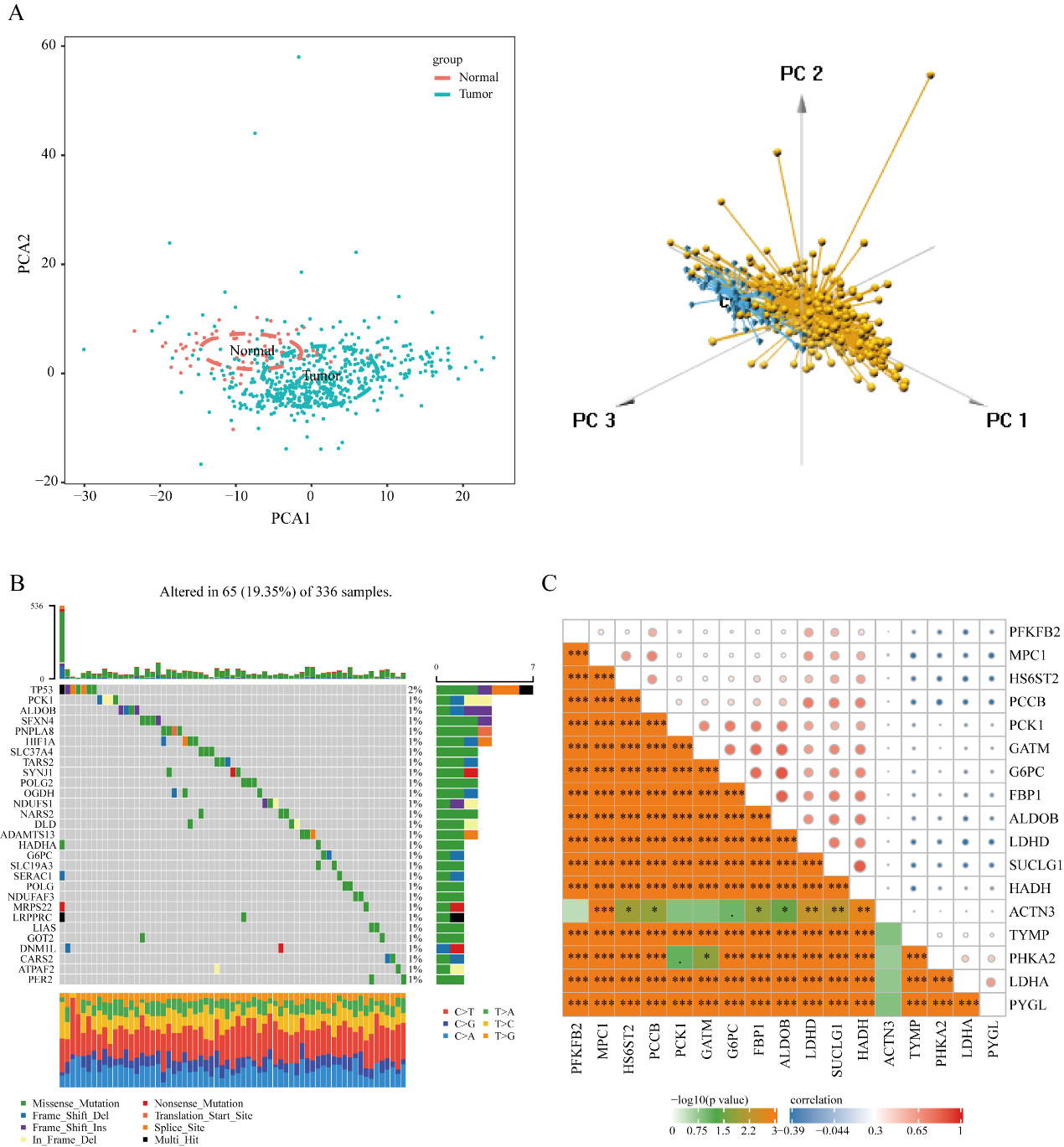


**Supplementary Figure 1.Multi-Omics Landscape of LRGs in KIRC.** (A) PCA and three-dimensional PCA for 267 LRGs, indicating significant different gene expression patterns in normal kidney and KIRC tissues. (B) The mutation frequency of 267 LRGs in 336 KIRC patients. (C) Expression correlations between 17 LRGs in the TCGA cohort using Spearman analyses. **P* <0.05, ***P* <0.01, ****P* <0.001.


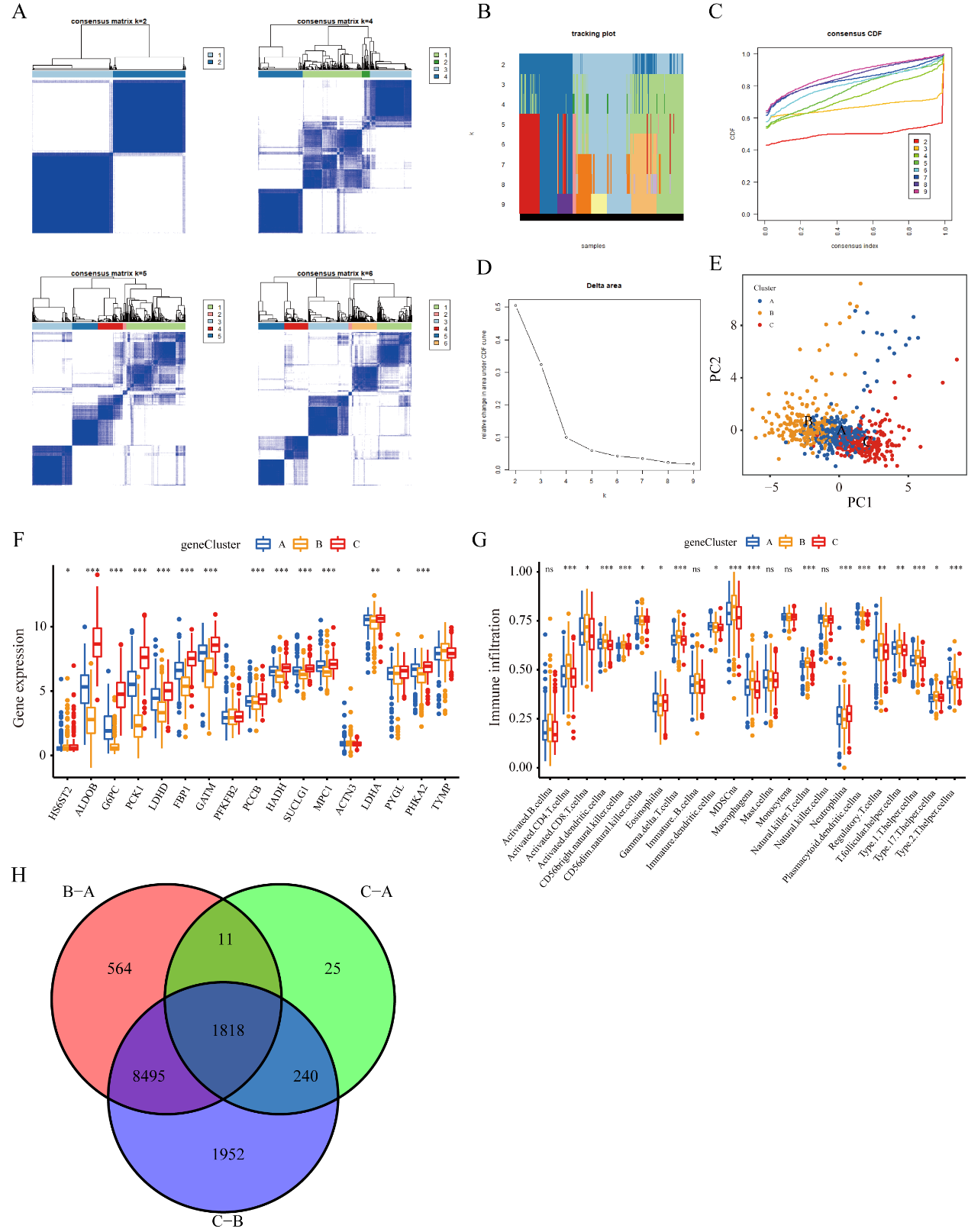


**Supplementary Figure 2. Unsupervised clustering of 17 differentially expressed LRGs in KIRC.** (A) Consensus matrices of the ACRG cohort for k = 2, 4, 5, 6. (B) The sample distribution changed with k valued 2 to 9. (C) Consensus clustering cumulative distribution function (CDF) with k valued 2 to 9. (D) The relative change in area under the CDF curve. (E) PCA for three clusters indicates significant different distribution patterns. (F) Boxplot shows the expression of 17 LRGs in the three clusters. (G) Boxplot shows the relative abundance of immune cell in the three clusters. (H) 1818 lactate phenotype-related DEGs shown in Venn diagram. **P* <0.05, ***P* <0.01, ****P* <0.001, ns: not significant.


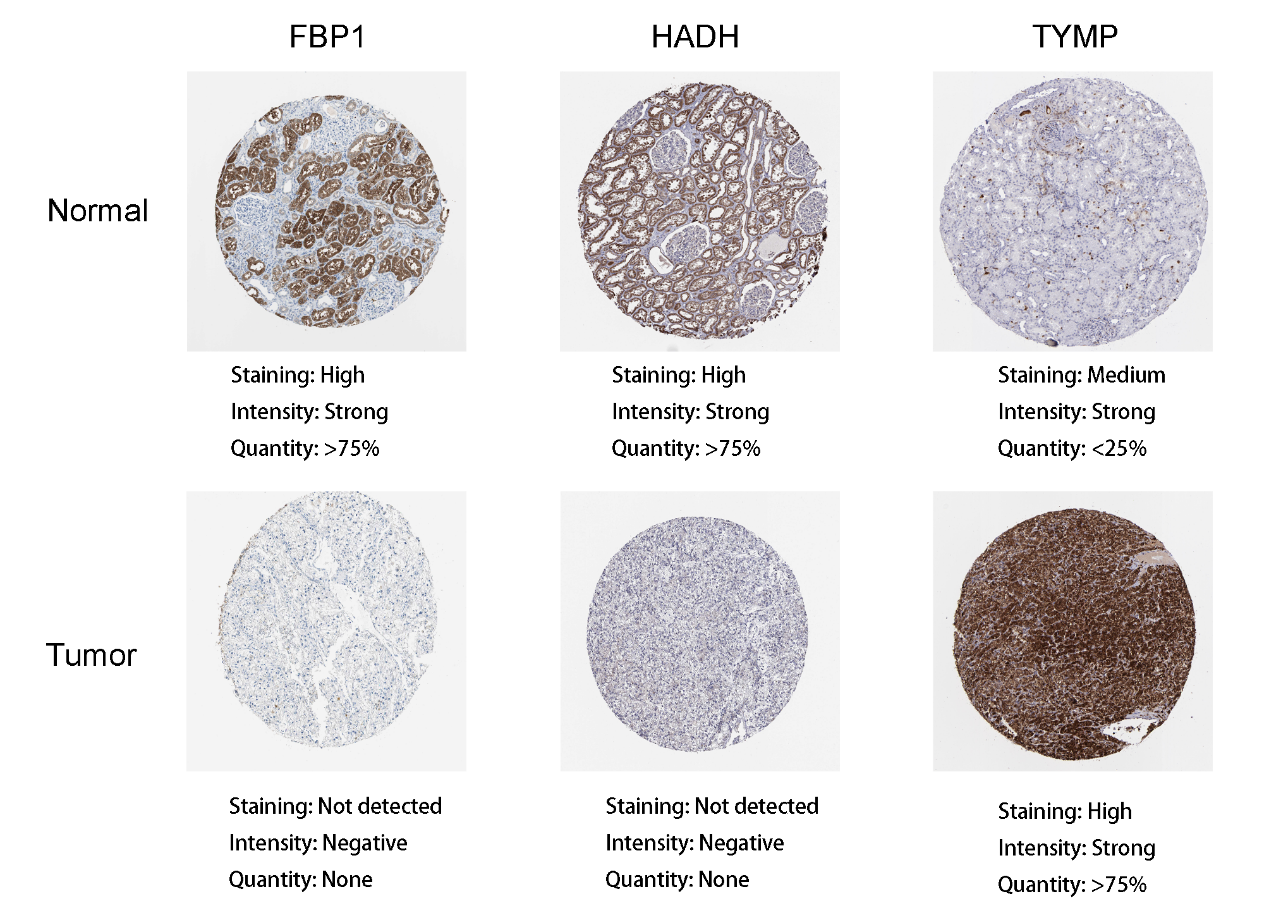


**Supplementary Figure 3. Immunohistochemistry of the six target genes based on the HPA database.**


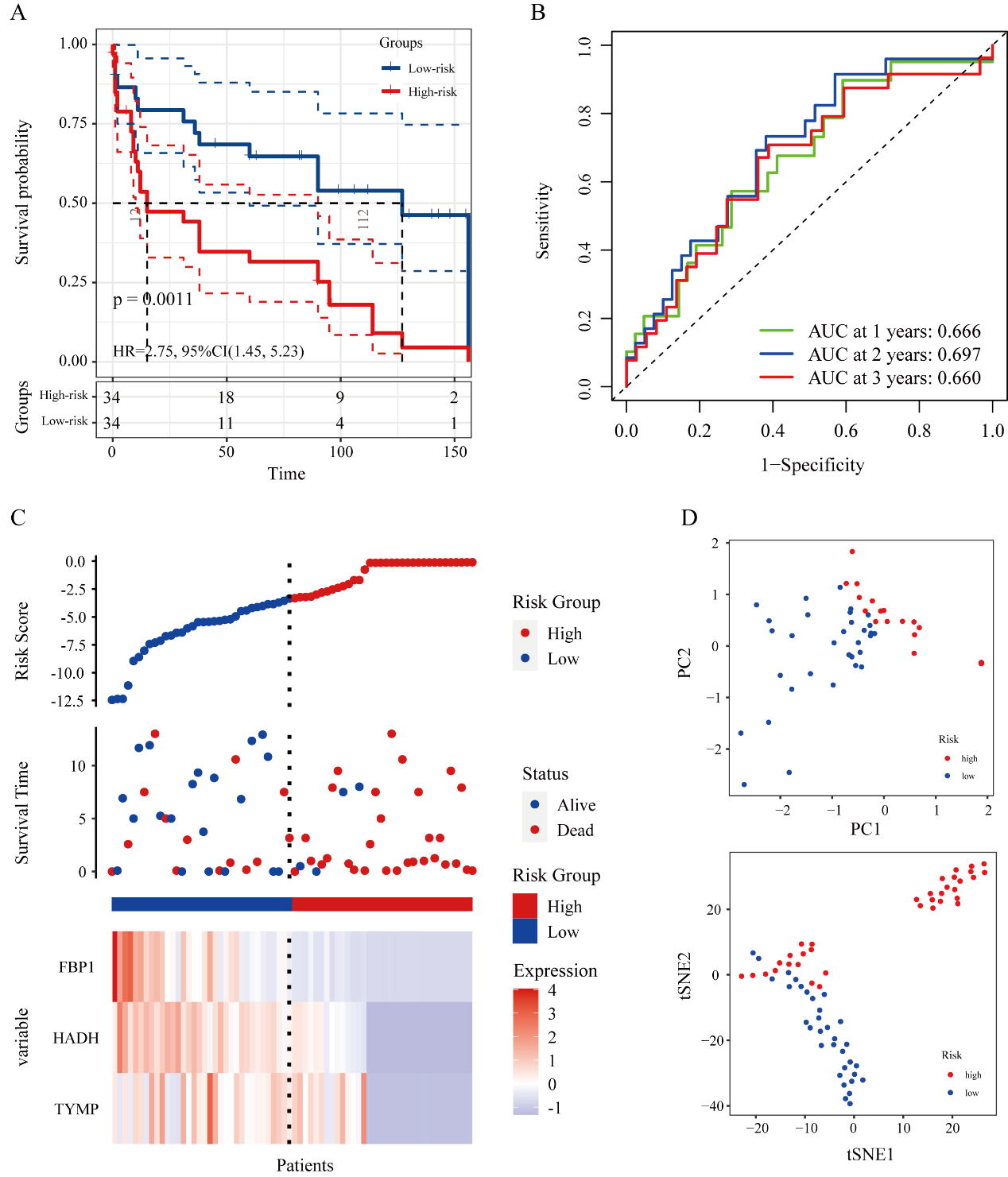


**Supplementary Figure 4. Validation of a lactate-related prognostic signature (****LRPS) in** **GSE22541 cohort.** (A) The Kaplan-Meier analysis of OS in the GSE22541 cohort. (B) ROC analysis of the LRPS in predicting 1-, 3-, and 5-year OS in the GSE22541 cohort. (C) The distribution plots of the risk score, OS status, and heatmap of gene expression in the GSE22541 cohort. (D) PCA and *t*-SNE analysis plot in the GSE22541 cohort.


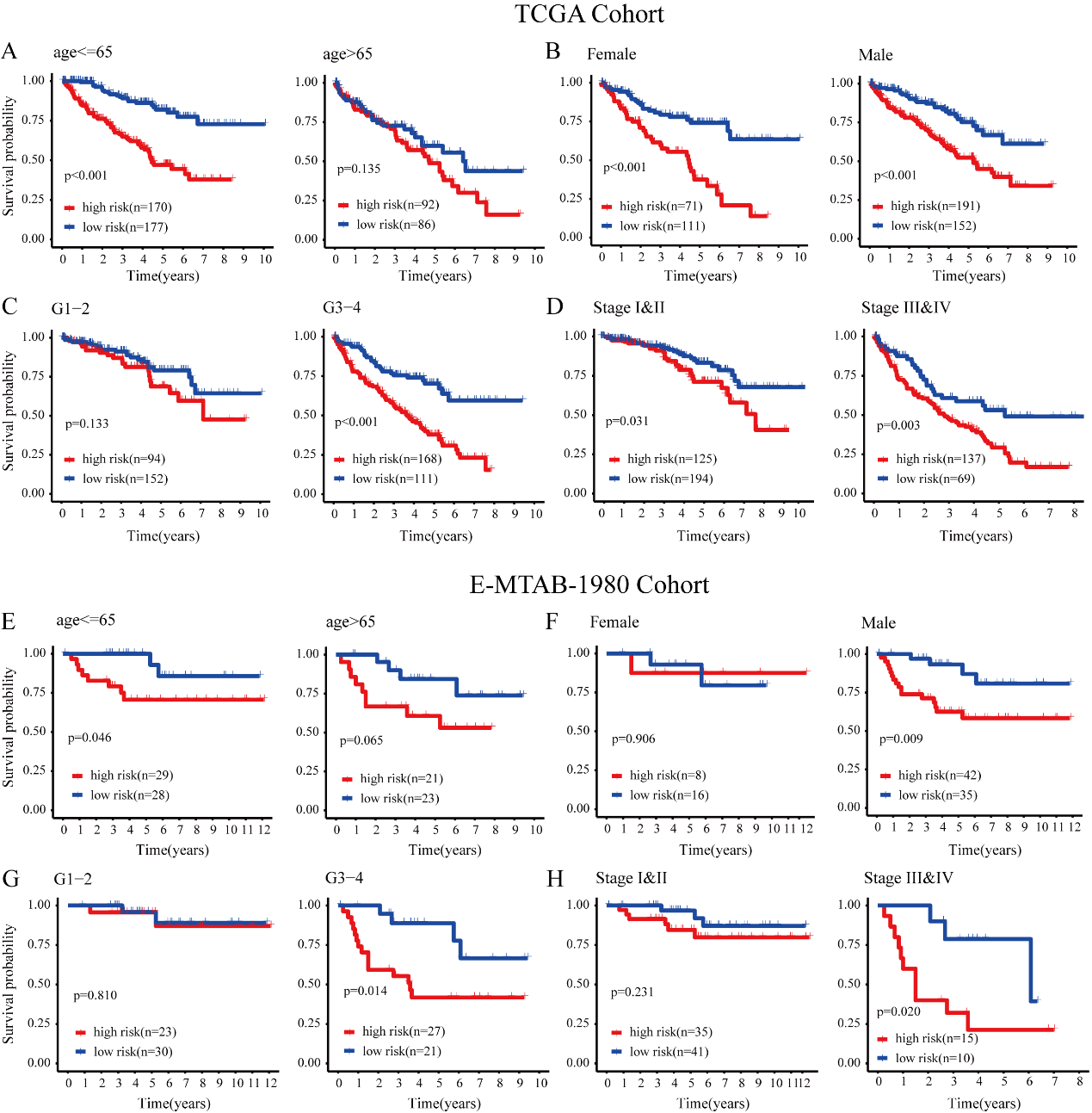


**Supplementary Figure 5. Kaplan-Meier curves of overall survival based on LRPS stratified by clinicopathologic risk factors.** Survival analysis of patients in low- and high-risk groups based on LRPS in different subgroups: age (A), gender (B), tumor grade (C) and stage (D) in the TCGA cohort. Survival analysis of patients in low- and high-risk groups based on LRPS in different subgroups: age (E), gender (F), tumor grade (G) and stage (H) in the E-MTAB-1980 cohort.


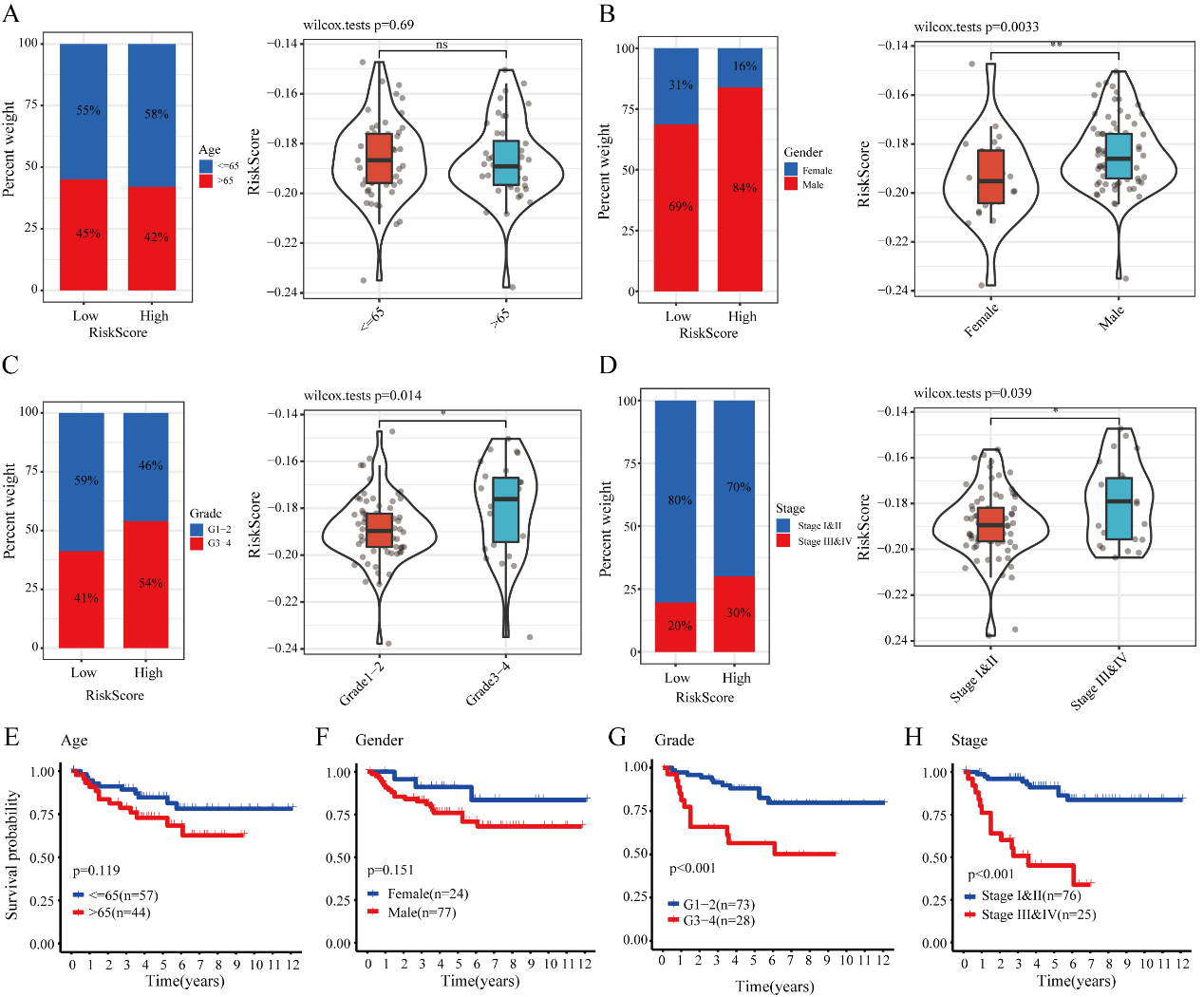


**Supplementary Figure 6.** **Correlation between LRPS and clinical features in** **E-MTAB-1980 cohort.** (A–D) The proportion of clinical features (age, gender, tumor grade and stage) in the low- or high-risk group. Distribution of risk scores in different groups according to clinical features. (E–H) Kaplan-Meier survival analysis for patients with KIRC based on the LRPS stratified by clinical features. **P* <0.05, ***P* <0.01, ns: not significant.


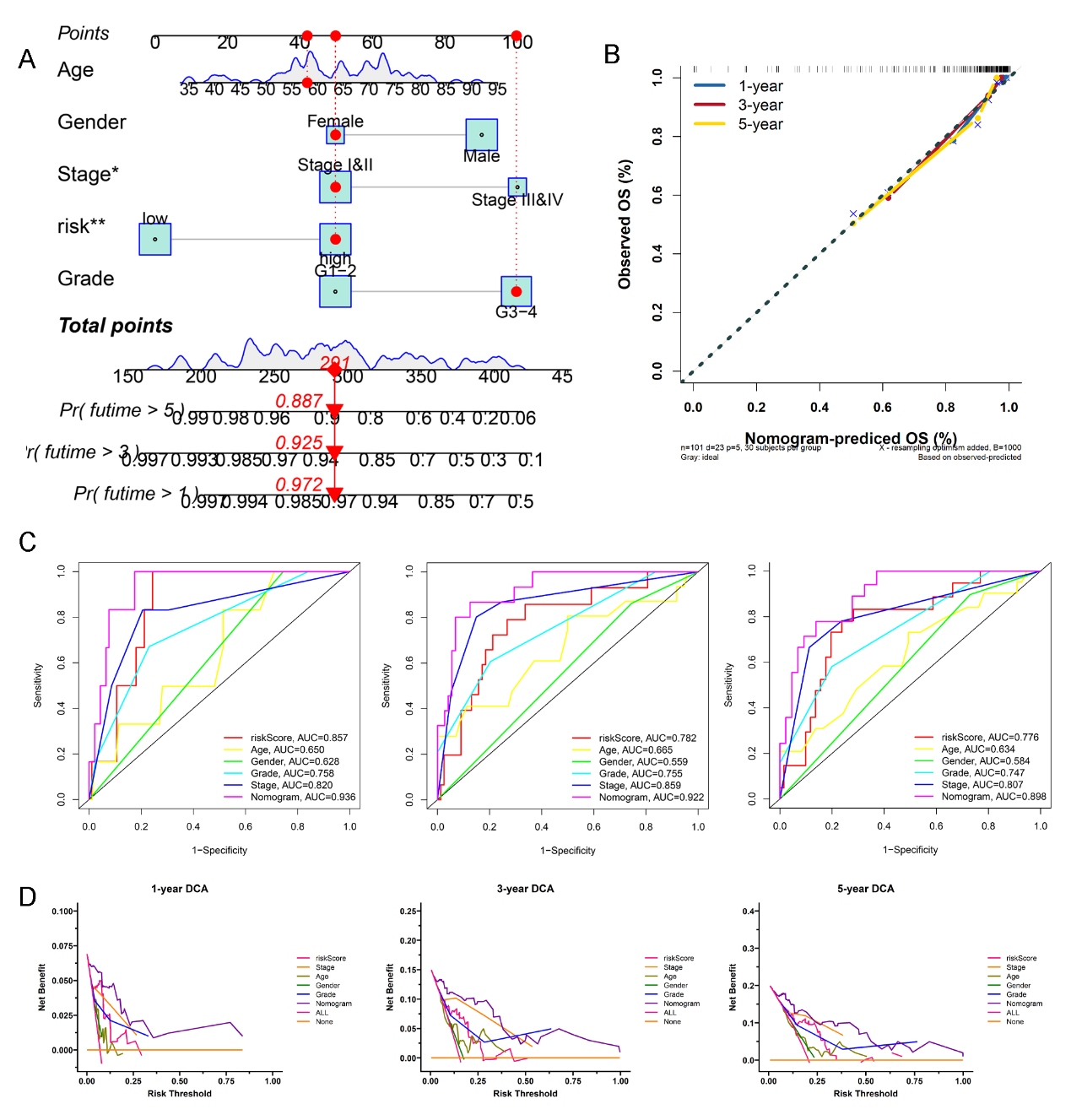


**Supplementary Figure 7.** **Construction of a nomogram predicting OS for patients with KIRC in E-MTAB-1980 cohort.** (A) Nomogram based on LRPS, age, gender, tumor grade and stage. (B) The calibration curves for internal validation of the nomogram. (C) Time‐dependent ROC curves of the nomogram in predicting 1-, 3-, and 5-year OS. (D) DCA curves of the nomogram.


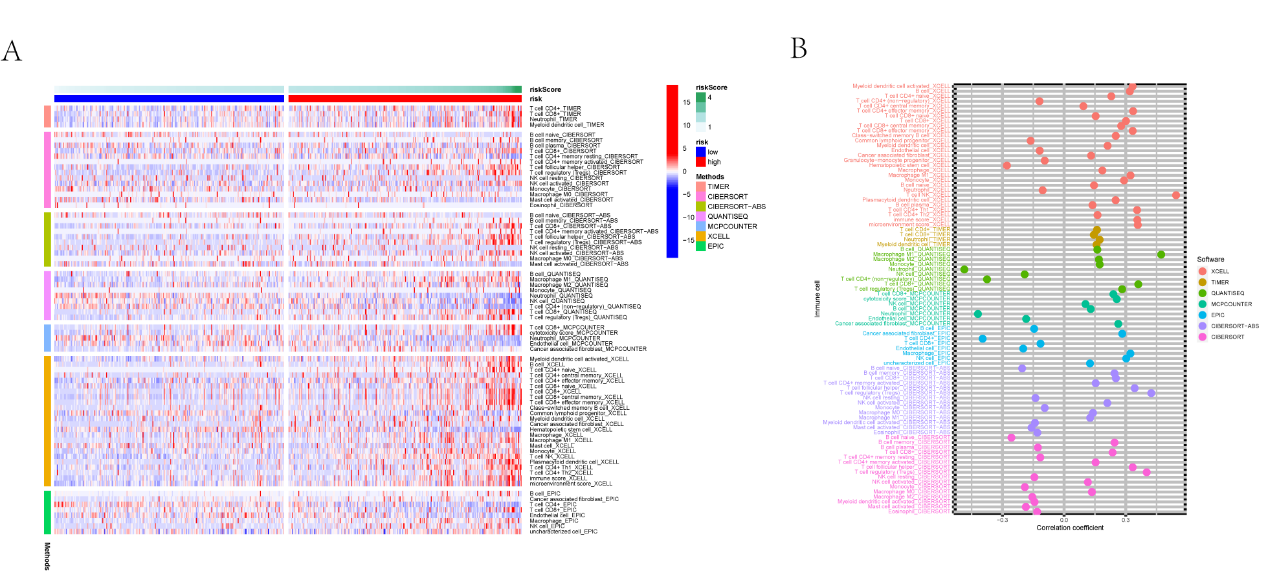


**Supplementary Figure 8. Estimation of tumor-infiltrating cells by the LRPS.** (A) Heatmap for immune responses based on TIMER, CIBERSORT, ESTIMATE, MCPcounter and ssGSEA algorithms among high and low-risk groups. (B) The lollipop plot shows of the correlation coefficients of the components with LRPS-based risk score.


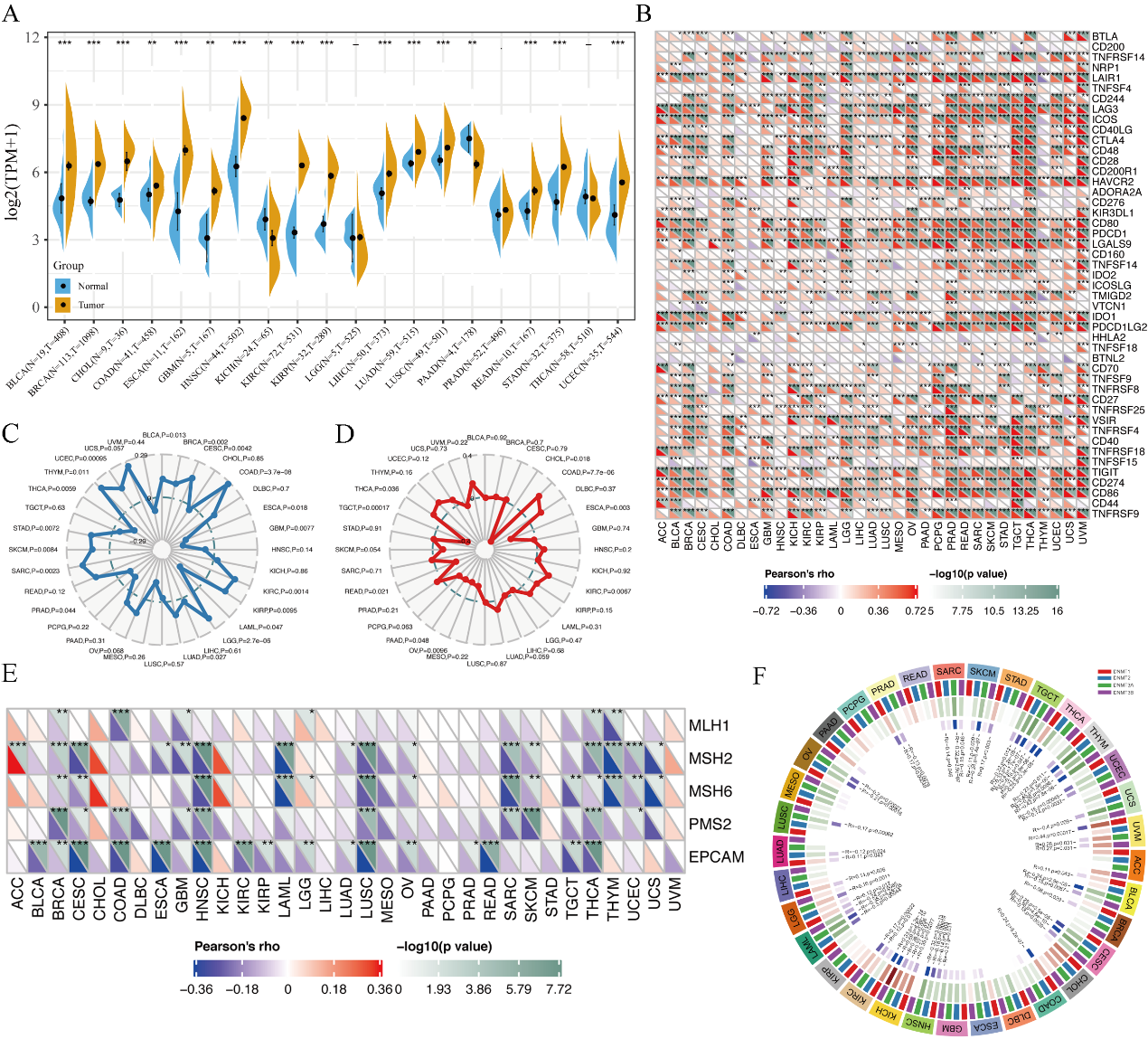


**Supplementary Figure 9.** **Pan-cancer analysis of the *TYMP*.** (A) TYMP expression in different types of cancer. (B) Heatmap of the association between TYMP protein levels and expression of immune markers across 33 cancer types. The correlation between TYMP expression and, TMB (C), MSI (D), MMR (E), and DNMT (F) across 33 cancer types. **P* <0.05, ***P* <0.01, ****P* <0.001.
